# Ways of Knowing Compassion: How Do We Come to Know, Understand, and Measure Compassion When We See It?

**DOI:** 10.3389/fpsyg.2020.547241

**Published:** 2020-10-02

**Authors:** Jennifer S. Mascaro, Marianne P. Florian, Marcia J. Ash, Patricia K. Palmer, Tyralynn Frazier, Paul Condon, Charles Raison

**Affiliations:** ^1^Department of Family and Preventive Medicine, Emory University School of Medicine, Atlanta, GA, United States; ^2^Graduate Division of Religion, Emory University, Atlanta, GA, United States; ^3^Department of Behavioral, Social, and Health Education Sciences, Rollins School of Public Health, Emory University, Atlanta, GA, United States; ^4^Department of Spiritual Health, Woodruff Health Sciences Center, Emory University, Atlanta, GA, United States; ^5^Center for Contemplative Science and Compassion-Based Ethics, Emory University, Atlanta, GA, United States; ^6^Department of Psychology, Southern Oregon University, Ashland, OR, United States; ^7^School of Human Ecology, University of Wisconsin–Madison, Madison, WI, United States

**Keywords:** compassion, empathy, altruism, methods, phenomenology, compassion meditation

## Abstract

Over the last decade, empirical research on compassion has burgeoned in the biomedical, clinical, translational, and foundational sciences. Increasingly sophisticated understandings and measures of compassion continue to emerge from the abundance of multidisciplinary and cross-disciplinary studies. Naturally, the diversity of research methods and theoretical frameworks employed presents a significant challenge to consensus and synthesis of this knowledge. To bring the empirical findings of separate and sometimes siloed disciplines into conversation with one another requires an examination of their disparate assumptions about what compassion is and how it can be known. Here, we present an integrated theoretical review of methodologies used in the empirical study of compassion. Our goal is to highlight the distinguishing features of each of these ways of knowing compassion, as well as the strengths and limitations of applying them to specific research questions. We hope this will provide useful tools for selecting methods that are tailored to explicit objectives (methods matching), taking advantage of methodological complementarity across disciplines (methods mixing), and incorporating the empirical study of compassion into fields in which it may be missing.

## Introduction

The last decade has seen a substantial increase in the empirical study of compassion. Programs of research investigate the phylogenetic continuity and evolutionary history of compassion (Goetz et al., 2010; Preston and Hofelich, 2012; Gilbert and Mascaro, 2017; Marsh, 2019), the physiological systems supporting compassion (Gilbert, 2014a; Kemper et al., 2015; Wang et al., 2019), and the impact of compassion on psychological and physical health (Galante et al., 2014; Neff et al., 2016). Along with this more foundational research, applied and translational studies examine the role and optimal manifestation of compassion in healthcare and educational settings, and test the efficacy of interventions and training programs aimed at expanding compassion toward self and others in a variety of contexts (McCaffrey and McConnell, 2015; Bibeau et al., 2016; Sinclair et al., 2016b; van Berkhout and Malouff, 2016; Luberto et al., 2018). Each of these domains of research has advanced in large part due to the development of measurement tools for identifying, describing, and quantifying compassion, as well as for empirically evaluating theoretical models of compassion. While this abundance of multidisciplinary and cross-disciplinary research has advanced what is known about compassion, the diversity of methods, assumptions, and theoretical frameworks makes it challenging to draw conclusions across studies and/or to incorporate compassion research into new fields, especially fields in which *compassion* may already be partially or implicitly operationalized.

While not without contention, large bodies of literature have generally cohered around a definition of *compassion* as a benevolent emotional response toward another who is suffering, coupled with the motivation to alleviate their suffering and promote their well-being (Dalai Lama, 2002; Goetz et al., 2010; Halifax, 2012; Klimecki et al., 2013; Post et al., 2014; Singer and Klimecki, 2014; Strauss et al., 2016; Sinclair et al., 2017c; Gilbert, 2019). From this starting point, we will survey research conducted on compassion and related constructs that share or resemble some or all of the basic criteria that characterize compassion. These are (1) an awareness of another’s suffering, (2) a benevolent emotional or affective response, and (3) the motivation to help or act (Strauss et al., 2016).

This theoretical review of empirical methods used to study compassion has the broad aim of promoting communication, collaboration, and convergence across disciplines. Our goal as a team of interdisciplinary scholars trained in foundational and applied areas of public health (K.P., M.A., and T.F.), social psychology (P.C.), biological anthropology (J.M. and T.F.), psychiatry (C.R.), and religious studies (M.F.) is twofold. First, we provide an integrated and interdisciplinary theoretical review of methods currently used in the empirical study of compassion. Second, we examine the strengths and limitations of applying them to specific research questions. We hope this will provide useful tools for selecting methods that are tailored to explicit objectives (*methods matching*), taking advantage of methodological complementarity across disciplines (*methods mixing*), and incorporating the empirical study of compassion into fields in which it may be absent or non-operationalized (*methods missing*) (for an overview of key terminology used in this article, see glossary in Table 1).

**TABLE 1:** A glossary of terms and their associated definitions used in this paper.

***Compassion***	– A benevolent emotional response toward another who is suffering, coupled with the motivation to alleviate their suffering and promote their well-being.
***Consilience***	– The convergence of evidence from independent sources and methods of measurement to support one coherent conclusion; connecting concepts, models, and principles from different disciplines to promote the formation of a unified and comprehensive theory.
***Convergence***	– When two separate processes produce similar results; unifying distinct technologies or bodies of knowledge and practice. In many ways the study of compassion is convergent because many separate disciplines have developed theories and methods for studying compassion or similar constructs, however, we also advocate deliberate effort to promote the unification of these somewhat siloed lines of inquiry.
***Empirical perspective***	– A basic structure inherent in all ways of knowing, which refers to the position[ality] or point-of-view of the informant reporting on compassionate phenomena, relative to the person or entity that is generating, experiencing, and/or enacting compassion.
***First-person perspective***	– A subjective or self-referential perspective, which illuminates a perceiver’s feeling/perception of their own compassion as it manifests to the self.
***Second-person perspective***	– An interpersonal perspective regarding indicators of another person’s compassion as it is directed toward the perceiver during an interaction.
***Third-person perspective***	– An objectifying perspective that sheds light on compassion data that as perceived by persons who are not involved in the compassionate experience or encounter being assessed.
***Frame of reference***	– An underlying, often shared set of assumptions, or conceptual relations that (at least partially) governs how empirical phenomena are perceived, categorized, and evaluated or how forms of evidence impact reasoned argument. Theoretical frames of reference may be context-specific and are subject to revision to increase their accuracy or broaden their applicability.
***Heuristic features***	– Key features that assist in the process of sorting various ways of knowing compassion into meaningful groups that can then be evaluated, combined, and/or applied to answer research questions.
***Method***	– A way of pursuing knowledge; an orderly, systematic procedure for obtaining data supporting or contradicting a claim.
***Methods-mapping***	– Formalizing correspondences between theoretical and methodological concepts in one research domain with one or more related concepts in another domain; this work proceeds on the presumption that shared or related meanings connect the concepts being mapped.
***Methods-matching***	– Selecting a method that allows the researcher to draw the most direct inferences based on the correspondence between (1) the data a method produces and (2) the specific question being addressed.
***Methods-missing***	– Addressing a compassion research question without using methods intended to assess compassion and/or without operationalizing the outcome as compassion.
***Methods-mixing***	– Coordinating different research methods by combining or juxtaposing them, in order to respond to research questions more completely, directly, and precisely.
***Ways of knowing compassion***	– Empirical methods and corresponding evidence that indicate compassionate affect and motivation may be present when an individual or group perceives suffering.

Within the scope of this review, we have deliberately set aside a number of worthwhile goals. First, we do not intend to critique alternate definitions or ways of operationalizing compassion. Constructive critiques are ongoing to refine and validate the construct of compassion, but this is not our project (Singer and Klimecki, 2014; Gu et al., 2017). Neither do we intend to privilege any empirical method or set of methods over others. For our purposes here, the suitability of a method is principally driven by research objectives. In addition, while many studies helpfully review and evaluate the impact of compassion (Eisenberg et al., 2010; Perrone-McGovern et al., 2014), these are too numerous and wide-ranging to adequately evaluate here. Moreover, this will not be a systematic or meta-analytic review of any one method. Our goal, instead, is to forge connections between disparate areas of compassion research in order to generate an overview of the current state of available methods for studying compassion. Lastly, we do not seek to prescribe directions for future research. Rather, we will conclude with recommendations for selecting and combining methods to advance understandings of compassion and maximize knowledge transfer across domains.

## Background

Research indicates that compassion has immediate health benefits for both the giver and receiver (Fogarty et al., 1999; Steffen and Masters, 2005; Galante et al., 2014), positively impacts relationship outcomes (Neff and Beretvas, 2013; Perrone-McGovern et al., 2014), and improves resilience in the context of adversity threat (Cosley et al., 2010; Neff and McGehee, 2010; Lim and DeSteno, 2016; Presnell, 2018). In medical care, compassion is linked with improved patient satisfaction, compliance, and clinical outcomes (Patel et al., 2019). In the workplace, compassion is associated with improved employee resilience and retention, as well as overall organizational health (Kanov et al., 2004; Spreitzer et al., 2013). In educational settings, compassion is associated with emotional well-being among children and adolescents (Neff and Pittman, 2010; Roeser and Eccles, 2015), and cultivating compassion during adolescence may lay the foundation for well-being during this sensitive period of social development and beyond (Játiva and Cerezo, 2014; Roeser and Pinela, 2014; Bach and Guse, 2015). Compassion also stands at the center of some third-wave psychotherapeutic interventions, which emphasize the relationship between thoughts and emotions (Gilbert, 2010, 2014b; Hayes and Hofmann, 2017). For example, compassion-focused therapy is an evolutionarily and neurophysiologically informed approach to psychotherapy that aims to improve mental health by understanding and promoting a compassionate motivational system (Gilbert, 2014b).

In many contexts, compassion is thought to be trainable either as a skill in itself or as an emergent gestalt of underlying skills that can be cultivated (Kanov et al., 2004; Klimecki et al., 2014). Motivated by this assumption, evidence-based training programs have proliferated for cultivating compassion for social and emotional health (Pace et al., 2009; Germer and Neff, 2013; Jazaieri et al., 2013; Roeser et al., 2018; Schuling et al., 2018; Borden, 2019; Condon and Makransky, 2020). Compassion has also emerged as a core value and “active ingredient” of diverse helping professions and professional environments, especially in medical care. At least 25 interventions have been developed to cultivate compassionate nursing care (McCaffrey and McConnell, 2015; Blomberg et al., 2016), and compassion training has become a more explicit goal of medical training and practice and is a key component of the American Medical Association’s first principle of medical ethics (Shih et al., 2013; American Medical Association, 2016; Rao and Kemper, 2017). In addition, in 2013, the Centers for Medicare and Medicaid Services implemented a value-based purchasing system that tied hospital reimbursement to patient satisfaction surveys, making patient-rated compassion critical to healthcare systems’ bottom line (Centers for Medicare and Medicaid Services (CMS), HHS, 2011).

While this breadth and depth of research on compassion and compassion training has arguably advanced scientific understanding and improved clinical, educational, and professional outcomes, there are several inherent issues complicating the study of compassion. First, because compassion includes both an affective and motivational component, there is a lack of consensus about how to compare and draw inferences from studies employing disparate units of measurement or levels of analysis. For example, recurring questions arise about relationships between behavioral and physiological observations on the one hand, and indicators of compassionate affect and motivation on the other: Can researchers intuit a compassionate state in the absence of physiological or behavioral data? Can researchers intuit a compassionate state from physiology or behavior *alone*?

Second, prominent models of compassion implicitly or explicitly assume that compassion emerges from discrete competencies, which can, in turn, be differentially facilitated or inhibited (Halifax, 2012; Lown, 2016; Gu et al., 2017). One influential evolutionary account theorizes that compassion is a suite of universal physiological and experiential responses that emerges because of situation-dependent cognitive appraisals. Besides the basic perception that someone is indeed suffering, compassionate responding is facilitated by the following appraisals: (1) the suffering individual is both relevant and of value to oneself; (2) the sufferer does not deserve their suffering; and (3) one is capable of helping (Goetz et al., 2010). The influence of this and similar models has propelled research focused on emotions and skills that may be necessary but incomplete constituents of compassion. Understanding complex interactions among these components requires empirical strategies that can differentiate between them and explore their dynamics.

Third, compassionate responses themselves are context-, experience-, and state-dependent, requiring empirical methods sensitive to factors ranging from bodily states to social and environmental conditions. A large body of theoretical and experimental research indicates that compassion is influenced by the observer’s perceptions of the in-group/out-group status of the suffering individual(s) (Cikara et al., 2011; Preston and Ritter, 2013). Such perceptions can depend on psychological resources (Dyrbye et al., 2019), environment (Kim and Lopez de Leon, 2019), psychological priming (Mikulincer and Shaver, 2001), and training or intervention (Kang et al., 2014). Understanding this broader picture of compassion, including psychological states and traits, relationships, environment, and personal history, is crucial for designing appropriate compassion research and for interpreting and contextualizing any findings.

Fourth, multiple related constructs, including but not limited to altruism, empathy, empathic concern, sympathy, prosociality, and care, overlap with broad understandings of compassion and should be considered part of the body of empirical knowledge about it. Significant obstacles to comparing data on compassion-related constructs arise because of well-documented shifts in how they are operationalized and defined (Batson, 2009; Marsh, 2019). Yet, their conceptual relatedness suggests that mapping—that is, formalizing and conventionalizing how terms in one research domain correspond with one or more terms in another field—could reveal that transdisciplinary findings converge in significant ways. Related, disparate fields of inquiry have distinct sets of methodologies, assumptions, and theoretical frameworks, which we will explore below. All of these inherent challenges invite consideration from those designing, interpreting, and evaluating research on compassion in any discipline.

We understand *ways of knowing compassion* to be any empirical phenomena that signal to an investigator that compassionate affect, motivation, and action are present in an individual or group. This includes signs that a necessary component of or condition for compassion may be present. Such an empirical approach to understanding compassion requires a consilient effort to alternate between vantages that focus on measurable physical, biological, and behavioral changes, and on more holistic vantages that focus on human-level, emergent properties of experience and interaction (Slingerland and Collard (eds), 2012a). Each way of knowing compassion that we describe evinces strengths and limitations. Some are more deeply shaped by the propensities of humans as social beings, including tendencies toward explanatory confabulation, concern for socially desirable self-representation, expectancy bias, memory bias, errors in affective forecasting, and plain old *not knowing*. Through understanding these, we can identify complementarity among different frameworks and methodologies and combine approaches and findings strategically to strengthen evidence and claims.

## Heuristics

**FIGURE 1:**
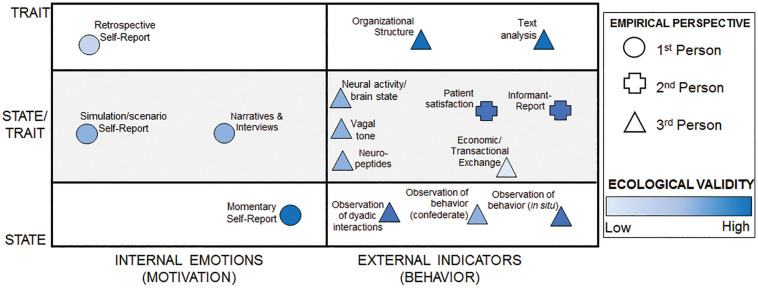
Mapping the ways of knowing compassion. This figure maps the major methodologies reviewed here into theoretical spaces. The shape of the methodology denotes frame of reference. Color represents the extent to which that method has ecological validity. Positioning on the *x*-axis corresponds to the extent to which a method measures internal versus external aspects of compassion. Positioning on the *y*-axis corresponds to whether the methodology is generally used to measure state or trait compassion or is used to measure both. Methods on the line between state and trait can be used to measure both, depending on the specifics of the methodology.

Among the ways of identifying and quantifying compassion, four clusters of features serve as guideposts or heuristics: (1) empirical perspective, (2) state versus trait, (3) quantitative versus qualitative, and (4) ecological validity. Figure 1 organizes the major methodologies reviewed according to these guiding heuristics.

### Empirical Perspective

To examine diverse methods for studying compassion, we will employ a heuristic feature related to the *empirical perspective* or *point of view* reflected in their evidence. That is, if there is a compassionate experience in question, it may be examined from a first-, second-, or third-person perspective. First-person data typically focus on the subjective experience and self-reported assessment of one’s own compassion, collected in scale questionnaires, interviews, and focus groups. Studies that rely at least partially on first-person perspectives collect participants’ reports on subjective experiences of compassionate feelings and motivations in response to others’ suffering. Methodologies rely on data emerging from first-person perspectives, when researchers collect, analyze, and interpret participants’ observations as primary evidence of compassion, or when study participants interpret their own experience of compassion as in phenomenological accounts. Second-person data often represent the perspective of the receiver or *in vivo* witness of compassion. Studies that depend on *second-person* evidence assess when and how participants recognize and experience compassion in others, be they companions, peers, caregivers, supervisors, or entire organizations. A third-person perspective, or observational perspective, applies when the experimenter or observer determines the presence, absence, and measurement of compassion, and interprets evidence such as physiological and behavioral observations. In this case, the observer neither experiences nor receives the compassion in question. These three perspectives can be mapped onto the *emic* and *etic* distinctions (Pike, 1967). Here, third-person perspectives emerge from an etic point of view: observations made by persons outside and relatively objective to the compassionate feeling, action, or interaction under study. First- and second-person perspectives arise from the emic point of view, provided by those who have an insider’s perspective on the compassion (or lack thereof). Of note, we use this heuristic differently than qualitative researchers, who often refer to the interviewee and interviewer using a first- and second-person distinction (Stelter, 2010).

Each empirical method or way of knowing compassion yields evidence from one or more of these perspectives and can be strategically selected to address the researchers’ questions, frameworks, or models of compassion. In other words, those interested in the internal thoughts or emotions surrounding compassion may be correct in prioritizing a first-person perspective. On the other hand, researchers interested in the behavioral aspect of compassion may be better served by informant-reporting and/or third-person measures (discussed below). Complementary first- and second-person measures may together create a more nuanced, accurate understanding of the relationship between internal states and external behavior. Moreover, combining self-report with second- or third-person reporting promises to generate new questions and hypotheses to explain conflicting evidence. In the main sections of this review to follow, we found *empirical perspective* to be a helpful superordinate criterion for organizing and presenting the various ways of knowing compassion.

### State Versus Trait

Another heuristic is the familiar psychological distinction between dispositional or trait-level versus momentary or state-level measurement. Many studies employ measures that frame compassion as a fluctuating internal *state*, and self-report is used to query the extent to which a respondent endorses feeling compassion at that point in time. In addition to self-report measures of compassionate states, researchers also detect compassion by observing behavior—including speech—that is best explained by the occurrence of a compassionate state, such as responding to another person’s suffering with demonstrable care or help (or expressing the desire to respond). These approaches investigate the relationship between internal processes and/or external circumstances and varying intensities of compassionate affect, motivation, and observable behavior.

Other research methods seek to understand compassion as an enduring individual or psychological *trait*. Traits, unlike states, are relatively stable aspects of a person’s way of thinking, feeling, and acting across time and in a broad range of circumstances. Because fluctuating conditions or contexts tend not to dislodge an individual’s traits, their origins or causes are, in theory, traceable to more stable and general underlying processes. This is not to say that traits are immutable or hardwired. Indeed, contemplative practices and other ways of priming and cultivating compassion usually presume that repeatedly engendering compassionate states will gradually strengthen the corresponding trait (McCrae and Costa, 1995; Baumert et al., 2017; Goleman and Davidson, 2017). Similarly, in the context of social and emotional education, traits are considered factors that have some level of mutability over child development (Knafo et al., 2008; Bengtsson et al., 2016). This view of traits is informed by Bandura’s (1976, 1999) impact on the field of behavioral learning, which posits that traits can be capabilities that are learned. From this perspective, compassion, like other social and emotional capabilities, can be cultivated over the course of child development and with training, an assumption that guides many social and emotional development programs. Some methods reviewed below aim to illuminate dispositional or trait compassion or the extent to which individuals tend to have compassion throughout their life.

### Quantitative Versus Qualitative

A third heuristic category that distinguishes ways of knowing compassion is the distinction between *quantitative* and *qualitative* methods. Quantitative data are numeric values that correspond directly or indirectly to measurements and/or observations of compassionate phenomena. Qualitative data, by contrast, describe compassionate phenomena in language or images to be interpreted using non-mathematical methods. While specific features of qualitative data, such as directions of change, intensities, frequencies, etc., can be systematically quantified, doing so rounds out potentially explanatory features and context that do not translate into numeric values (Gavin, 2008; Ruane, 2016). Merging two of the heuristics that we will use here, all three empirical (first-, second-, and third-person) perspectives can be queried using quantitative and qualitative methods.

### Ecological Validity

Lastly, ways of knowing compassion generate data that vary in *ecological validity*, meaning that they cannot be uniformly transferred or generalized from controlled settings to real-life contexts outside the research setting. Theoretically, the more closely a study’s methods mirror everyday life, the more ecologically valid their evidence will be. Usually, studies with stricter control of variables sacrifice this advantage in favor of precision, replicability, or other strengths. Ecological validity is an especially weighty consideration in light of the social and environmental situatedness of emotions and the centrality of emotion, in the form of affect and motivation, to our understanding of compassion and how it manifests (Griffiths and Scarantino, 2009).

## Ways of Knowing Compassion

### First-Person Perspective

In this section, we begin our review of ways of knowing compassion with research methods for collecting and analyzing first-person empirical evidence, including quantitative and qualitative approaches to understanding compassionate states and traits.

#### Quantitative

Self-report measures that use first-person data to quantify compassion are the most common methodological tools researchers use, particularly in the health and psychological sciences (Sinclair et al., 2017c), and are by far the most common outcome measures used in randomized controlled trials to assess the impacts of interventions for increasing compassion and prosociality (Luberto et al., 2018). The majority of self-report measures assess compassion as a dispositional or trait-like quality. One example, the Compassionate Love Scale (Sprecher and Fehr, 2005), rates 21 items reflecting two subscales: compassion toward significant others (example item: “If a person close to me needs help, I would do almost anything I could to help him or her”) and compassion toward strangers or humanity more widely (example item: “When I see people I do not know feeling sad, I feel a need to reach out to them”).

Self-report measures of the *absence* or *inhibition* of compassion are arguably more developed within the literature than measures of compassion itself. These compassion-negative constructs include empathic distress,^1^ burnout, compassion fatigue, and secondary traumatic stress. They indicate conditions in which a potential caregiver fails to experience or exhibit compassion. The implicit and sometimes explicit explanation is that the caregiver’s reserves of compassion are depleted and/or displaced by feelings of frustration, emotional isolation, exhaustion, and a decreased sense of accomplishment and meaning (Boyle, 2015). Compassion fatigue has been studied among caregivers and providers who work in stressful environments and who are frequently exposed to suffering and death, including physicians, nurses, first responders, teachers in at-risk school districts, and spiritual caregivers (Roberts et al., 2003; Yoder, 2010; Hotchkiss and Lesher, 2018; Buelher, 2019). In healthcare, the Professional Quality of Life Scale is frequently used to examine the relationship between compassion fatigue, burnout, and secondary traumatic stress among providers (Alkema et al., 2008; Smart et al., 2014; Beaumont et al., 2016). While the construct of compassion fatigue receives frequent attention, critical reviews of this area highlight the need for further research that explicitly addresses the relationship between failures of compassion and compassion itself (Fernando, and Consedine, 2014; Ledoux, 2015; Sinclair et al., 2017b). Measurement will be integral toward this end.

Whether quantifying compassion or its absence, self-report measures have various limitations (Strauss et al., 2016). Many commonly used scale questionnaires are retrospective in nature, meaning they require participants to summarize their experience over an entire day, week, month, or a lifetime (e.g., “How much stress have you felt over the past 2 weeks?”; Conner and Barrett, 2012). These retrospective measures tend to reflect participants’ beliefs about themselves rather than their actual behavior, lived experience, or physiological correlates (Mauss and Robinson, 2009). Relatedly, in simulation or hypothetical scenario-based questionnaires, participants may be asked to recall or imagine a helping scenario, rate their sense of compassion, and speculate about whether they would help. Responses in this paradigm are most likely driven by generalizations about the self (e.g., “I am a compassionate person”) and about the value of specific emotions and helping behavior (e.g., “Compassion leads to helping, which feels good.”). People often underestimate or overestimate how they might feel in a hypothetical circumstance, which is known as a limitation in *affective forecasting* (Wilson and Gilbert, 2003). For instance, physicians’ reports of their probable experience of compassion in response to hypothetical vignettes might not resemble their actual interactions with patients. Further complicating matters, the self-reported experience of an emotion does not always match prototypical conceptions of emotional experiences, for example, when fear feels pleasant during a scary movie. This mismatch has been shown to be true of compassion in particular, with study participants reporting that compassion prototypically feels uniformly pleasant yet describing both pleasant and unpleasant experiences of compassion (Condon and Barrett, 2013).

Because of limitations of retrospective self-reports, many researchers rely on momentary measures, often classified as *ecological momentary assessment* or *experience sampling* techniques. These techniques require participants to carry a device, such as a smartphone, and respond to alerts or prompts in the moment throughout their day (e.g., “How much compassion do you feel toward your patients?”). Studies have shown that such measures are more closely associated with real-time physiology and behavior patterns than retrospective self-report measures (Conner and Barrett, 2012). This technique has not been widely applied to the study of compassion; however, one experience sampling study demonstrated that compassion meditation training resulted in reduced momentary reports of mind-wandering and corresponding increases in self-reported caring behaviors (Jazaieri et al., 2016). While findings from momentary assessment have high ecological, convergent, and predictive validity, they are time- and resource-intensive. Moreover, although momentary reports overcome some of the limitations of retrospective reports, they remain subject to social desirability and participant expectations, although likely to a lesser degree.

#### Qualitative

Qualitative, first-person methods based on narratives, interviews, interactions, or focus groups examine the richer contours of compassion. These approaches allow participants to contextualize their responses, appraise significance, and inform researchers about unexpected factors that arise *in situ*. They capture first-person experiences and interpretations, although not exclusively. To analyze the complexity of narratives, dialog, and descriptions requires rigorous planning, often relying on computer-assisted qualitative data analysis software (Lewins and Silver, 2007; Saldaña, 2011, 2016).

Qualitative descriptive (QD) research uses a variety of forms of data, including first-person accounts, to craft a detailed description of a situation or process and suggest further avenues of inquiry (Sandelowski, 2000; Leeman et al., 2007; Kim et al., 2017). This method has been used to investigate experiences and causes of compassion fatigue among nurses (Berg et al., 2016; Fukumori et al., 2018). Often, QD research is an initial step before more controlled and fine-grained experimentation and analysis (Neergaard et al., 2009).

Grounded theory is a more methodologically formal procedure for analyzing qualitative data, which is used in the human, social, and health sciences. It involves time-consuming recursive sifting, categorizing (i.e., coding), and interpretation to discover recurring themes and patterns in participants’ responses and interactions (Bryant and Charmaz, 2007). To understand compassion, grounded theorists examine firsthand accounts of participants’ perceptions and/or experiences by reviewing and sorting transcribed interviews and interactions to identify themes or patterns that recur throughout a data set and code passages of text exemplifying those themes. They then interpret the prevalence and significance of recurring themes and features (for examples, see Crowther et al., 2013; McPherson et al., 2016; Sinclair et al., 2017a; Tierney et al., 2017; Jain et al., 2019). Many grounded theory accounts focus exclusively on respondents’ conceptual understandings of compassion and may not assess any specific occurrence of compassion. Such projects help constitute a way of knowing how compassion is understood by a person or group. In general, grounded theory is best suited to exploratory projects that supplement or pave the way for explanatory studies (Bryant and Charmaz, 2010).

Other qualitative research in the human and social sciences relies on a phenomenological framework for collecting and analyzing first-person data (Dowling, 2007). This approach takes inspiration from the philosophical phenomenological tradition initiated by Edmund Husserl and developed by subsequent phenomenologists interested in developing a rigorous “descriptive psychology” of conscious phenomena such as existence, perception, care, and empathy (Husserl, 1989; Stein, 1989; Fisette, 2018; Zahavi, 2018). From its inception, phenomenology arguably launched the first-person empirical study of compassion-related experiences. Phenomenological method involves systematically altering one’s attitude toward one’s own perceptions and cognitions, which permits a more rigorous and systematic study of subjective states. By investigating how different phenomena appear to conscious awareness, phenomenologists seek to discover an underlying structure governing consciousness itself.

However, philosophically trained phenomenological researchers are quick to note that the majority of phenomenology-inspired scientific studies depart significantly from foundational methods and questions and are conspicuously unconcerned with investigating the structure of consciousness (Giorgi, 2010; Smith, 2016). Phenomenology-inspired empirical studies of compassion address questions ranging from how participants identify subjective experiences of feeling, receiving, and training in compassion (Pauley and McPherson, 2010), to what compassion “is like for them” to experience, receive, and cultivate (Lawrence and Lee, 2014; Jarvis, 2017). Other studies address similar questions regarding compassion inhibition, fatigue, etc. (Waite et al., 2015; Jack, 2017).

All qualitative first-person evidence has the potential to reveal insights into how compassion is conceived of and experienced firsthand and how conscious, subjective understandings, and attitudes lead to compassionate behavior. For example, qualitative approaches have documented the uniquely rewarding and replenishing feelings that can be associated with compassion, even in the face of suffering, a documented experience of highly trained contemplative practitioners (Dreyfus, 2001). First-person perspectives also reflect human sensitivities to social desirability, usually framed as an evaluative bias, which is the tendency to present oneself in a positive light and potentially underreport socially undesirable thoughts or behaviors. The presence of an interviewer often increases social desirability biases, an effect that can be moderated by the gender and characteristics of the respondent (Krumpal, 2013). Qualitative researchers have given rigorous thought to minimizing social desirability biased responding, especially in interviews about highly evaluative topics (Fisher, 1993; Johnson and Van de Vijver, 2003; Bergen and Labonté, 2020). While subjective, qualitative accounts of compassion draw connections between experiences, interpretations, and acts of compassion, findings are often not intended to be generalizable or transferable to different groups and settings. Still, it is clear that first-person data can reveal otherwise unknowable information about the mental contents of the compassionate (or non-compassionate) individual being studied. In this way, first-person data can also be used to complement second- and third-person empirical perspectives.

### Second-Person Perspective

The limitations inherent to first-person reports of such a highly evaluative construct as compassion highlight the importance of verification with other empirical perspectives. Methods examining second-person evidence of compassion, also referred to as *informant reporting*, is one approach for doing so. Examples of informant reports of compassion include teacher reporting on children’s compassion, often using a psychometric instrument such as the Prosocial Behavior subscale of the Teacher Social Competence Scale (Harter, 1982). Other informant reports measure compassionate acts within an intimate relationship, for example, Reis et al. (2014)’ 10-item dyadic inventory of compassionate acts.

Informant reporting by medical patients is a common method for assessing healthcare provider compassion (Sinclair et al., 2017c). Scale questionnaires measure general state-level compassion conveyed in a particular clinical encounter. Examples of such tools include the Physician Compassion Questionnaire (Fogarty et al., 1999), the Compassionate Care Assessment Tool (Burnell and Agan, 2013), the Schwartz Center Compassionate Care Scale (Lown et al., 2015), and a new 5-item clinician compassion measure (Roberts et al., 2019). Healthcare provider compassion is also measured by informant reports from colleagues in both allopathic and osteopathic medicine (Evans et al., 2004), as well as clinical psychology (Kaslow et al., 2009).

Some widely used measures of patient satisfaction in healthcare assess general aspects of care that are understood to tangentially reflect patient experiences of compassionate care. The Press Ganey patient satisfaction survey includes items assessing the degree to which hospital staff “addressed your spiritual needs” and “addressed your emotional needs.” One study of more than 1.7 million patient responses observed that ratings of how well staff addressed patients’ spiritual and emotional needs correlated with three Press Ganey performance areas: (1) staff response to concerns or complaints, (2) staff effort to include patients in treatment decisions, and (3) staff sensitivity to the inconvenience that health problems and hospitalization can cause (Clark et al., 2003). The Consumer Assessment of Healthcare Providers and Systems, a patient satisfaction measure widely used in Medicare and Medicaid value-based purchasing, has versions for hospital (H-CAHPS) and outpatient (CG-CAHPS) contexts (Centers for Medicare and Medicaid Services (CMS), HHS, 2011; Dyer et al., 2012). One cross-sectional study of 269 acute care hospitals in the United States found that hospitals that reward provider compassion and provide compassionate support for their employees have higher H-CAHPS ratings and are more likely to be recommended by patients (McClelland and Vogus, 2014). The H-CAHPS survey has also been used to examine compassion in the context of a hospital chaplain consultation by measuring elements of the interaction commonly understood to comprise compassionate care (Marin et al., 2015).

Qualitative research methods are also used to examine compassion from second-person perspectives. Indeed, this method may be a particularly apt alternative or complement to the measurement of overt or external behavior and its impact (Vazire and Mehl, 2008). In-depth interviews allow participants to report on the importance and meaning of receiving compassion, specifics that could not be anticipated in a survey question and that may not translate into quantitative measurement. In their exemplary study, Sinclair et al. (2016a) interviewed 53 palliative care patients and used grounded theory to analyze their experiences of providers’ compassion. They also compared these experiences of compassion with patients’ experiences of related constructs, such as *empathy* and *sympathy* (Sinclair et al., 2017a). They found that patients viewed overt behaviors such as demonstrative and grandiose expressions of emotion as emblematic of *sympathy* and reported it as off-putting. In contrast, patients saw subtle behaviors, often falling outside of routine care and tailored to individual needs, as authentically compassionate (Sinclair et al., 2017a). The resultant empirical model of compassion is arguably the most comprehensive in clinical medicine. It identifies provider virtues such as authenticity, tolerance, and honesty as essential ingredients of compassion, and it details how these requisites of compassion are carried out in a clinical relational context.

While these strengths may tempt us to conclude that informant reports are inherently more reliable and powerful than self-reports of compassion, it is important to consider the potential sources of explicit and implicit bias when using second-person compassion data, just as with first-person data. Again, our point is not to discourage the use of any research method, but rather to assist in strategic use of multiple research methods to gain a clearer understanding of compassionate phenomena. First, it is likely that informant reports of compassion are skewed by cultural and class differences, as well as racial and gender biases, similar to those shown to impact informant reporting of other non-compassion behaviors and competencies (for example, in student evaluations, Fan et al., 2019). There is, moreover, some evidence to indicate that such biases may influence perceptions of care received from out-group members. For example, one study found that patient–provider social concordance levels (a measure of the patient and provider’s match on race, gender, age, and educational status) were related to patient ratings of satisfaction with their provider’s care (Thornton et al., 2011). Therefore, rather than ranking the value of any one perspective on compassion, we believe that matching methods and perspectives to the research questions they are best suited to answer is vital, as we will discuss below.

### Third-Person Perspective

A broad array of methods and evidence are used when observing compassion from a third-person point of view. In fact, any quantitative and qualitative data can be studied from a third-person standpoint, even when the evidence itself reflects participants’ subjective experiences of extending and receiving compassion. The crucial difference lies in whether data are examined for their insights into the subjective perception, experience, or understanding of compassion, or whether data are being marshaled as intersubjective evidence of compassion itself. In this review, we do not intend to overlook the ways that third-person observers’ subjective tendencies influence their findings and conclusions. This undoubtedly influences all research on compassion. However, we distinguish empirical perspectives as third-personal by emphasizing how the object of inquiry is specified, while remaining cognizant that there will be overlap and ambiguity in specific cases. Third-person evidence may include researcher’s observations of human-, animal-, and group-level behavior and functioning, as well as measurements of physiological changes from which compassion might be inferred, such as brain states, facial expressions, writings, etc. Human-made products—discourse, design principles, art, laws, archeological, and other artifacts—can also serve as intersubjective evidence of compassion. In the following section, we discuss several forms of third-person evidence from which a state or disposition of compassion may be inferred.

#### Compassionate Behavior

A great deal of behavioral research on compassion is conducted using social psychology experimental methodologies. Social psychologists generally view compassion as a prosocial state that is responsive to others’ suffering and that motivates costly helping behaviors intended to alleviate suffering, potentially at the expense of oneself. An action or state is *prosocial* to the extent that it is conducive to social bonding and acceptance. While prosocial helping is distinct from compassion, it is understood as an outcome of some compassionate motivational state. As such, costly helping behavior is often used to infer that compassion is present. For this reason, observations of helping behaviors have been instrumental in garnering ecological validity for compassion as a psychological construct that can influence human (and perhaps animal) behavior. Batson et al. (1983) pioneered several paradigms for studying costly helping in which participants observe a *confederate*—an actor posing as a study participant—typically facing a difficult situation, such as receiving electric shocks or experiencing distress over a car crash or academic demands. Importantly, these paradigms are constructed such that self-interested factors such as seeking social recognition and avoiding punishment could not explain the participant’s decision to engage in the costly helping behavior. Participants who opt to help are therefore thought to be demonstrating a compassionate state (Batson et al., 1991; Batson, 2009; Goetz et al., 2010).

Confederate paradigms that assess prosocial behavior in real-time settings are perhaps the criterion standard for ecologically valid prosociality research—they overcome limitations of self-reports because of memory and affective forecasting biases and provide direct assessment of actions that alleviate others’ suffering in situations that reflect daily life. In this way, researchers can measure prosocial behavior when participants themselves are not aware that they are being observed. At the same time, confederate paradigms can be difficult or inefficient to implement, given that they require careful training of confederates and careful debriefing to assess participant suspicion. Additionally, some research scenarios may skew behaviors in a prosocial direction. For example, a participant might demonstrate compassion for someone receiving shocks or struggling with academic work within a confederate paradigm but may not be able to access or extend compassion as readily in a familiar context. Intriguingly, experiments using confederate scenarios have demonstrated the efficacy of mindfulness and compassion training for enhancing prosocial behaviors, even when situational pressures dampen the impulse to help, such as offering one’s seat to a stranger who is using crutches, even when others seated nearby are unresponsive and ostensibly less considerate (Condon et al., 2013).

Other research in social psychology has used both naturalistic and simulated settings to demonstrate positive changes in real-world prosocial behavior after various types of meditation training across different contexts. In one study, mindfulness training was associated with participants’ increased willingness to interact with an ostracized individual via Cyberball, a computer-based ball-tossing game, an effect that was mediated by self-reported warmth and compassion (Berry et al., 2018). Compassion training was also associated with reduced amygdala reactivity and more sustained visual attention to scenes of suffering in an experiment using an eye-tracking protocol (Weng et al., 2018). In another experiment, compassion training was associated with greater increase in participants’ optimism and willingness to write a letter to a convicted murderer (Koopmann-Holm et al., 2019). Behavioral markers of compassion in naturalistic settings, much like confederate-paradigm studies, can require extra time and resources to capture and evaluate, yet they reveal diverse genres of compassion-evincing behaviors across contexts and populations.

As an alternative to confederate and other behavioral paradigms, researchers often use controlled economic exchanges to examine generosity and cooperation in monetary transactions. Various studies have demonstrated that kindness-oriented meditation programs enhance prosocial behavior in the form of economic donations. Loving–kindness meditation has been shown to enhance prosocial helping in computer-based video games (Leiberg et al., 2011) and in online economic transactions (Weng et al., 2013, 2015). Among preschoolers, a mindfulness-based kindness curriculum resulted in increased peer donations of stickers (Flook et al., 2015). Economic paradigms have also been fruitful in neuroimaging studies that link compassion-related neural processes with prosocial behavior (Leiberg et al., 2011; Weng et al., 2015; Ashar et al., 2016). While behavioral economic measures offer a well-controlled environment for research on prosocial behavior and are widely used for studying influences on human cooperation and moral decision-making, they are often conducted via computer-based interfaces and impose artificial constraints on social exchange. This approach lacks ecological validity with respect to real-time face-to-face social interactions. Results likely reflect distinctive psychological dynamics of exchange relationships that may not apply to the social bonds that occur in close communal relationships (Clark and Mils, 1993). It is unclear to what extent economic generosity extends to common real-world situations involving the suffering of another individual that would purportedly elicit compassion (e.g., an interaction with a student who is struggling or a patient who is sick).

An alternative to experimental behavioral paradigms such as the confederate or behavioral economic approaches described above are naturalistic observational methods that increase ecological validity and reduce evaluative biases. One example is the Electronically Activated Recorder (EAR), an audio recorder that intermittently captures ambient sound throughout a person’s daily routine without the person being aware of when it is recording, yielding an acoustic log of the person’s day (Mehl, 2017). Previous studies have used the EAR to examine fathers’ empathic language and compassionate responses to their child’s cries (Mascaro et al., 2017). Another study used the EAR to examine correlations between (1) participants’ self-reported mindfulness and (2) language and behavioral indicators associated with mindfulness (Kaplan et al., 2018). The authors found that self-reported mindfulness was not related to prosocial behavior as assessed by the EAR, highlighting the kind of mismatch that can occur between different empirical perspectives (first- vs. third-person). To our knowledge, few studies have explicitly used the EAR to study compassion *in the wild*, and it remains a methodological tool of relatively high and untapped potential. While naturalistic observations offer high levels of external and ecological validity, they often generate a wealth of data and are time consuming to code and evaluate. In addition, they may be prohibitive in contexts where privacy and confidentiality are at a premium, for example, in clinical contexts.

#### Compassion in Dyads

Some third-person methods assess compassionate responding by evaluating a dynamic encounter between two or more people, such that the measurement takes into account the interchange between individuals. In the field of family psychology, researchers investigate dyadic behavior between parents and children or between intimate partners. A standard experiment involves having a parent and child collaborate on a difficult task. Researchers code and quantify communication and behavioral indicators that convey warmth (e.g., affection, encouragement, etc.) or that lack warmth (e.g., criticism, eye rolling, etc.) (Miller et al., 2015). Paradigms such as these can be used to couple personal, interpersonal, and physiological correlates with parental compassion (Miller, 2018). For example, Leerkes et al. (2016) examined mothers’ physiological arousal and behavior in response to a distressed infant, with a focus on sensitivity (e.g., appropriate calming behavior) and lack thereof (withdrawing). Methods such as this have been used to examine the impact of life history or trauma exposure on maternal caregiving behavior that occurs in the context of a mother–infant dyad (Strathearn et al., 2009). While the behaviors and constructs examined in these studies are often referred to as something other than compassion (e.g., parental warmth), from our perspective there is a great deal of overlap between these concepts and the model of compassion as an affective and motivational response to perceiving another’s suffering. We believe these findings will converge with those of related disciplines explicitly studying compassion.

Because compassion contributes to success in clinical encounters, third-person behavioral observations are also used to evaluate and understand compassion in these dyadic encounters. Interactions between patients and providers are either observed or recorded, and those data were analyzed using a variety of approaches (e.g., grounded theory). For example, Suchman et al. (1997) examined transcripts of clinical interactions for patients’ emotional expression (direct or implied) and corresponding physician responsiveness. Others have used an ethnographic observational approach and qualitative analysis to examine compassionate communication in hospice, in which the researchers provided a rich description of hospice workers engaging in emotion recognition, relating, and reacting to alleviate patient suffering (Way and Tracy, 2012).

A dyadic approach avoids many of the limitations and biases inherent in the use of self-report questionnaires. It also yields more ecologically valid findings than many behavioral paradigms, and dyadic analysis is a particularly useful tool to understand how compassion unfolds verbally or non-verbally among individuals. However, dyadic approaches are not without limitations. Of primary concern is a lack of agreement regarding the optimal markers or exemplars of compassionate behavior. For example, what constitutes compassion in a provider–patient interaction? Across studies examining patient–provider communication, a diversity of linguistic and performative markers have been coded as compassion (Beck et al., 2002). Common themes included reassurance, active listening, and responsiveness to emotional cues, yet consensus is lacking. Finally, if compassion requires an affective response and motivation to help, as is suggested by most definitions, then all observable behavior, whether occurring in dyads or not, must assess compassionate intentions primarily by inference.

#### Organizational Compassion

Emergent features of communities and organizations constitute yet another way of knowing compassion. In an influential article, Kanov et al. (2004) define organizational compassion as a collective noticing, feeling, and responding to suffering that promotes healing. They argue that organizational compassion differs from individual-level compassion in that it is collective, sanctioned, promoted, or codified by organizational norms and policies and then coordinated and propagated across individuals. Cameron and others likewise differentiate research investigating the culture and functions of an organization itself (“virtuousness *through* organizations”) from studies focused on individuals acting compassionately within an organizational context (i.e., “virtuousness *in* organizations”) (Kanov et al., 2004; Dutton et al., 2006; Cameron, 2017). Of the former, empirically tractable factors such as shared values, shared beliefs, norms, practices, leaders’ behavior, and the structure and quality of relationships relate to and indicate the emergence of organizational compassion (Lilius et al., 2008; Dutton et al., 2014; Cameron, 2017).

#### Physiology and Compassion

Detectable changes in the functioning and structures of the body are alternative ways of knowing compassion. In general, this physiological frame of reference rests on the tenet that brain and body systems are shaped by natural selection to engender compassion and related prosocial emotions and skills. A second tenet is that these states are associated with outward compassionate behavior. It follows from these assumptions that physiological assessment helps us understand the body’s necessary conditions and likely outcomes of compassion, as well as individual variation. In addition, there is often an implicit or explicit claim that physiological measures, not being subject to self-report biases described above, are inherently more accurate than other measures (Kirby et al., 2017).

The neurophysiological domain advances our ability to describe and quantify the activity of neural systems involved in compassion using neuroimaging assessment tools such as functional magnetic resonance imaging (fMRI) (Kim et al., 2020a), high-density electroencephalography and event-related potentials, and transcranial direct current stimulation (tDCS) (Petrocchi et al., 2017b). A common method involves inducing the affective components of compassion in participants using emotionally evocative picture or video stimuli of suffering others and comparing this putatively compassionate neural response to that which occurs while viewing neutral stimuli or stimuli thought to elicit other emotions, such as pride (Simon-Thomas et al., 2011; Klimecki et al., 2012). Other studies have examined the relationship between prosocial behavior during an economic game and neural activity elicited by compassion-inducing stimuli (Weng et al., 2013). Still other neurophysiological studies also look for correlations between participants’ self-reported state-level compassionate affect and neural activity elicited by a compassion-inducing task (see for example, Marsh et al., 2014; Brethel-Haurwitz et al., 2017). Other studies have examined brain function during the self-directed cultivation of compassion, for example, during compassion meditation (Engström and Söderfeldt, 2010; Schoenberg et al., 2018) or after compassion meditation training (Mascaro et al., 2013a, b). Findings from these assessments are inherently constrained by the relative paucity of ecological validity that can be achieved in a scanner environment, the inferences necessary to link behavior with internal compassionate states, and biases inherent in self-reports. Notably, a recent meta-analysis found some inconsistency in the existing findings on the neural correlates of compassion, especially with respect to the amygdala and midbrain regions important for pain modulation and autonomic function, which may relate to whether the compassion in question was generated as a “top-down” or “bottom-up” process. While there was a high degree of consistency in other brain regions thought to be important for compassion (anterior cingulate cortex, bilateral anterior insula, basal ganglia, and bilateral inferior frontal gyri), this meta-analysis pointed to a relative sameness in the methods used thus far to study compassion in the fMRI scanner. The researchers ultimately advocated increased specification of research targets and additional innovative methods to advance neurophysiological understandings of compassion (Kim et al., 2020a). Future research that combines multimodal physiological assessments will be informative for potentially providing convergent evidence about the bidirectional associations between multiple physiological systems important for compassion (e.g., see Nguyen et al., 2016; Petrocchi et al., 2017b; Kim et al., 2020b). Moreover, future studies combining neuroimaging assessments with behavioral and experience sampling methods will extend the ecological validity, precision, and discriminant validity of existing measures of compassion.

A related physiological methodology focuses on the role of neuropeptides thought to be important modulators of compassion. Oxytocin is a neuropeptide synthesized in the paraventricular and supraoptic nuclei of the hypothalamus and stored and released back into the brain and into peripheral circulation by the pituitary gland. Thus, oxytocin acts as both a hormone and a neuropeptide and has effects on both the brain and the body. Two decades of research have focused attention on the role of oxytocin in parental attachment and bonding, as well as in prosocial emotions, motivations, and behavior more broadly (Bethlehem et al., 2013; Johnson and Young, 2017). For example, Palgi et al. (2014) conducted a double-blind, crossover experiment in which participants self-administered either intranasal oxytocin or a placebo before listening to stories of suffering and writing compassionate responses to the victims in each story. The presence of self-administered oxytocin was associated with more compassionate responses toward women but not toward men. Other groups have examined the relationship between endogenous oxytocin and the amount of compassion participants report receiving or experiencing toward others. For example, endogenous oxytocin levels are positively correlated with the amount of maternal compassion that patients with bipolar disorder report receiving as a child (Ebert et al., 2018).

Other researchers have examined the possibility that autonomic responses to suffering, and their downstream impact on heart rate and breathing, can serve as a bodily signal of compassion. Porges’ polyvagal theory posits that, in the face of another’s suffering, an initial fight–flight response has to be down-regulated via myelinated vagal efferent pathways of the parasympathetic nervous system. Vagal tone, as the activation of these pathways is sometimes called, impacts cardiac function and the hypothalamic–pituitary–adrenal axis to support “spontaneous social engagement” in the face of distress by dampening other, less prosocial responses (Porges, 2007). Early research in this area highlighted the measurement of heart rate variability (HRV) as an indicator of parasympathetic activity. HRV is a measurement of the beat-to-beat changes in cardiac output, and early thought was that the ratio of high-frequency (HF) to low-frequency (LF) HRV reflects the intrinsic balance between parasympathetic and sympathetic activity. However, more recently, researchers have called into question whether the ratio of HF HRV to LF HRV is an accurate metric for the ratio between sympathetic and parasympathetic activity and identified alternate calculations of vagal tone as a more accurate reflection of the underlying physiology (Heathers, 2014). HF HRV and the root mean-square of successive differences have both been used in recent research as a measure of autonomic control of the heart, mediated by the vagus nerve (Matos et al., 2017; Petrocchi et al., 2017a; Kim et al., 2020b).

As recent critiques have improved the rigor of research using HRV as an index of vagal tone (Heathers et al., 2015), accumulating evidence supports the measurement of HRV for understanding and evaluating compassion. Researchers have found that HRV relates to the experience of compassion and predicted compassionate behavior (Stellar et al., 2015). Others have found that compassionate responses appear to rely on the parasympathetic nervous system to modulate the emotional response to suffering, as indexed by HRV (Rockliff et al., 2008). Still others have found that training in compassion meditation or engaging with compassion-focused therapy improves HRV, either during a resting state (Matos et al., 2017; Kim et al., 2020c), in response to stressful stimuli or a task (Petrocchi et al., 2017a; Ceccarelli et al., 2019), or during compassion training itself (Kim et al., 2020b). While not explicitly investigating compassion, another recent study used tDCS applied near the left anterior insula and found that stimulation increased both self-reported soothing positive affect and HF HRV. This innovative methodological approach links a brain region hypothesized to be important for compassion and empathy to both compassion-related affect and changes in HRV (Petrocchi et al., 2017b). Based on these findings, some have argued that HRV should be included as a primary outcome measure when assessing and training compassion (Kirby et al., 2017), and recent meta-analytic evidence supports this approach (Di Bello et al., 2020).

Other researchers have used the Facial Action Coding System (FACS) to quantify the spontaneous expression of compassionate affect elicited by video stimuli (Baránková et al., 2019). One of the first uses of this methodology emerged in a study of adults and children whose facial movements were documented as they watched a compassion-inducing video (Eisenberg et al., 1989). Researchers found that movements indicating “concerned attention” or “sympathy-directed toward another” correlated with later helping behavior. Compassionate facial movements included lowered and/or furrowed eyebrows, lowered upper eyelids, and sometimes raised lower eyelids, facing forward, and relaxation of the lower face and jaw. Another group used FACS to evaluate physiognomic responses to video stimuli of human suffering to determine whether responses were impacted by a 3-month meditation retreat (Rosenberg et al., 2015). They found that the intensive meditation training increased facial displays of sadness and decreased displays of rejection (operationalized as anger, contempt, or disgust). Of note, a recent theoretical article by Barrett et al. (2019) is skeptical of facial indicators of emotion, arguing that people do not express emotions with enough consistency or specificity to allow for the kinds of inferences made from FACS assessment. Moreover, even among prominent emotion scientists who endorse the theory that a core set of emotions has discrete biological bases—often referred to as “basic emotions”—a large majority (80%) do not believe compassion to be a discrete emotion (Ekman, 2016).

#### Compassion in Text

Other methodologies are used to qualitatively mine textual content for elements of compassion. Some researchers have used qualitative analysis of content from online platforms such as Facebook or Twitter to look at compassionate language and activity within a Facebook support group (Pounds et al., 2018) or by soliciting Twitter users to describe instances of organizational compassion toward healthcare staff (Clyne et al., 2018). As with non-virtual interactions, online communities can be analyzed at the individual or dyadic (and beyond) level, which has the potential to reveal the dynamic nature of the digitally mediated expression and reception of compassion (Sun, 2019). Others have conducted archival text analysis, for example, analyzing first- and second-century medical writing for evidence of physician compassion (Porter, 2016), or used exegetical and hermeneutic approaches to sacred texts to derive doctrinal or personal positions on compassion (See for example Sears, 1998; McCaffrey et al., 2012; Gibson, 2015; T̈āhir ul-Qādrī, 2015). While textual analysis has many of the strengths of the third-person perspective, one must consider the source of the text, which in some cases may be self-reported or informant-reported and therefore subject to the limitations of those methodologies.

## Summary and Conclusion

In this review, we have surveyed a variety of indicators and measures that have been used to define and study compassion. Examining these methodologies in the context of one another is vital to making compassion research more accurate, reliable, and transferable. It is also key for increasing knowledge transfer across the range of academic disciplines and other fields of compassion inquiry. Compassion is a multifaceted, intersubjective object of inquiry, glimpsed from a variety of separate viewpoints, each of which contributes to the unity of knowledge about compassion. We end with three summary points:

### Method Matching

First, we find it evident from this review that the method(s) chosen to evaluate compassion should be theory-grounded and guided by specific research hypotheses. There may be times when first-person self-report measures are the best choice; however, those should be privileged only when the person’s internal states are most crucial to the hypothesis being tested and with recognition of the limitations of this methodology. Similarly, it stands to reason that other hypotheses will require methods that tap other perspectives and frames of reference. For example, identifying facilitators and inhibitors of helping behaviors directed toward strangers would be most directly inferred from third-person (i.e., behavior-based) evidence rather than self-report.

We also suggest that more thought is warranted on the use of state measures of compassion when testing hypotheses about trait compassion. Behavioral and confederate paradigms are frequently used to measure changes in *trait* compassion, for example, after a compassion-training intervention. The underlying rationale is that one’s augmented compassionate trait makes it more likely that they will enter into a compassionate state, such that measuring the likelihood of a compassionate response tells us something about trait compassion. The relationship between trait and state compassion is of great interest to many, and more methodological sensitivity toward this issue will be important toward advancing the field of compassion science.

### Method Missing

In addition, our review process showed that certain research areas that target compassion would benefit from measurement techniques that are more fine-grained and that explicitly assess compassion. Some K–12 education programs explicitly target compassion cultivation as a broader focus, yet the majority of the effectiveness studies that provide the evidence base for such programs do not assess changes in compassion as a primary outcome being measured (Jones et al., 2017). This lack of explicit measurement makes it difficult to meaningfully evaluate whether compassion-based interventions targeting K–12 students actually promote the development of compassion. Given the demonstrated impact of compassion cultivation on resilience in adulthood (Bach and Guse, 2015; Bluth et al., 2016), education research explicitly assessing compassion in childhood and adolescence is well-warranted. Relatedly, the field of social and emotional education development could greatly benefit from interdisciplinary collaborations to create such measures.

It is also clear that there is a lack of clarity about how to measure compassion at the level of organizations and communities. Do the three core components of compassion—awareness of suffering, an affective response, and a motivation to help—also hold for organizations and communities? If so, what do “awareness” and “affective response” look like at the community or organizational level, and how can it be measured?

We have made the claim here that discipline-specific constructs such as *parental warmth* share a conceptual relatedness with compassion, such that cross-disciplinary sharing may reveal convergences. While this idea has in part motivated the current review, we view this claim as an empirical question for future research. Thus arise questions such as “What does the construct of parental warmth share with compassion for those who are unrelated?” We acknowledge that questions like these are not new (e.g., see Swain et al., 2012), but we contend that they will be informed by increased sharing of methods across disciplines. Of note, given the problematic history of the conflation of terms and constructs across disciplines, such work will require care and precision so as not to cause further confusion.

### Method Mixing

A key point that emerges from this review is the importance of strategic method mixing for studies of compassion. The multiple frames of reference we have discussed can be combined to create a more accurate understanding of the relationship between *internal* emotions, goals, and perceptions on the one hand, and *external* behavior on the other. There are valuable exemplars of method mixing already in the literature. For example, Sinclair et al. (2017a, 2018) used second-person qualitative evidence to understand the perspective of patients receiving compassion and then conducted a follow-up study to understand healthcare providers’ first-person experiences offering compassion. We are optimistic that future research across disciplines will continue to utilize method-mixing approaches; however, it is important to note that at times the results of such method mixing may contradict one another. In fact, this may be important in its own right. The resulting ambivalence can be addressed by enhanced research methods that combine and cross-reference multiple ways of knowing, such as correlating individuals’ self-report scale measures with their behavior, with informant reports, or by using neurophenomenological experimental designs. For example, within intimate couples, first- and second-person reporting could be combined to reveal discrepancies between the way compassion was intended and the way it was received. It is exactly this type of method mixing that has been called for in compassion neuroimaging studies, where researchers have argued that including measurements of both motivation and action in research on the physiology of compassion will be crucial toward establishing links between neurobiology, emotion, and behavior outside the laboratory (Kim et al., 2020b).

Moreover, method mixing could advance consensus within controversial areas such as *self-compassion* and *compassion fatigue* research. We believe combinations of first-, second-, and third-person compassion measures would help solidify our understanding of how compassion for self relates to compassion for others (López et al., 2018). In clinical research, method mixing can inform how obstacles to provider compassion relate to compassion failures and in so doing will provide a more nuanced landscape for identifying organizational solutions and interventions. Progress here will move the field beyond vague and abstract notions of compassion fatigue resulting from a depleted compassion reservoir and toward a richer understanding of the contexts and resources that foster sustainable compassion. Increasing the versatility and eclecticism of compassion research is of critical importance to comprehensive and interdisciplinary examinations of diverse ways of knowing compassion.

### Limitations

Our intent in this review was to summarize the current state of methodologies that are used to understand and quantify compassion across widely varying fields of inquiry. No doubt we bring our own disciplinary biases to this work, but throughout we have used this space to bridge disparate fields. These biases may have led us to overlook important methods that could have further enhanced this review. Moreover, while we defined *compassion* in accordance with our own disciplines, there are nuanced differences in how compassion is operationalized that will influence the methods chosen to study it. Because of issues of feasibility, while we attempt to incorporate disparate fields of compassion research, we were unable to review all areas to the same degree as the literature from psychology, religion, and contemplative science, with which we are most familiar.

### Conclusion

We contend that a better understanding of ways of knowing compassion is a type of consilience that at its best can improve research design, unify knowledge, and bridge disciplines for the benefit of all investigators interested in compassion (Wilson, 1999; Slingerland and Collard, 2012b). Future research will advance our knowledge by innovating novel ways of combining the measurement of multiple indicators of compassion. Ultimately, research designs that link the affective, cognitive, and motivational components of compassion with compassionate behavior will be of benefit to the many clinical, education, organizational, and interpersonal domains in which compassion is so critical to positive outcomes.

## Author Contributions

JM, MF, MA, PP, TF, and PC conceived of the manuscript and wrote significant sections. All authors provided critical and substantive feedback and critical revisions for important intellectual content.

## Conflict of Interest

The authors declare that the research was conducted in the absence of any commercial or financial relationships that could be construed as a potential conflict of interest.

## Footnotes

1 The Interpersonal Reactivity Index’s Empathic concern subscale is well-established and often equated with compassion [Davis, 1983. Measuring Individual-Differences in Empathy—Evidence for a Multidimensional Approach. *Journal of Personality and Social Psychology*, *44*(1), 113–126. <Go to ISI>://A1983PY32000010]. In fact, it includes items related to compassion, warmth, sympathy. The IRI is probably the most frequently used self-report measure in social psychology and closely related fields. It also has an Empathic Distress subscale.
